# Administration of Purified Alpha-1 Antitrypsin in Salt-Loaded Hypertensive 129Sv Mice Attenuates the Expression of Inflammatory Associated Proteins in the Kidney

**DOI:** 10.3390/biom15070951

**Published:** 2025-06-30

**Authors:** Van-Anh L. Nguyen, Yunus E. Dogan, Niharika Bala, Erika S. Galban, Sihong Song, Abdel A. Alli

**Affiliations:** 1 Department of Medicine, Division of Nephrology, Hypertension, and Renal Transplantation, University of Florida College of Medicine, Gainesville, FL 32610, USA; nguyenlva@yahoo.com (V.-A.L.N.); yunusemredogan@yahoo.com (Y.E.D.); niharikabala@ufl.edu (N.B.); egalban@ufl.edu (E.S.G.); 2 Department of Physiology and Aging, University of Florida College of Medicine, Gainesville, FL 32610, USA; 3 Department of Pediatrics, Faculty of Medicine, Erciyes University Kayseri, Kayseri 38039,Türkiye; 4 Department of Pharmaceutics, University of Florida College of Pharmacy, Gainesville, FL 32610, USA; shsong@ufl.edu

**Keywords:** alpha-1 antitrypsin, kidney, inflammation, 129Sv mice

## Abstract

Background: Alpha-1 antitrypsin (AAT) is a multifunctional protease inhibitor that has been shown to have anti-inflammatory properties in various diseases. AAT has been reported to protect against renal injury via anti-apoptotic, anti-fibrotic, and anti-inflammatory effects. However, its role in mitigating renal inflammation and reducing high blood pressure induced by salt-loading has never been studied. Methods: In this study, we salt-loaded 129Sv mice to induce hypertension and then administered purified human AAT (hAAT) or the vehicle to investigate whether renal inflammation and associated inflammatory/signaling pathways are mitigated. Results: Western blotting and densitometric analysis showed administration of hAAT attenuated protein expression of kidney injury molecule-1 (KIM1), CD93, CD36, and the toll-like receptor 2 and 4 (TLR-2/4) in kidney lysates. Similarly, protein expression of two key inflammatory transcription factors, signal transducer and activator of transcription 3 (STAT3) and NF-Kappa B were shown to be attenuated in the kidneys of 129Sv mice that received hAAT. Conversely, hAAT treatment upregulated the expression of heat shock protein 70 (HSP70) and immunohistochemistry confirmed these findings. Conclusions: Purified hAAT administration may be efficacious in mitigating renal inflammation associated with the development of hypertension from salt-loading, potentially through a mechanism involving the reduction of pro-inflammatory and injury-associated proteins.

## 1. Introduction

Hypertension is a major global health problem affecting more than 1.3 billion adults worldwide [1]. The association between hypertension and salt intake is well known and numerous studies highlight the impact of dietary sodium on blood pressure (BP). Excess salt consumption is a major contributor to hypertension, a global health problem that significantly increases the risk of cardiovascular diseases such as heart attack and stroke [2].

Damage to renal structures and dysregulation of proteins in different cell types in the kidney have been seen during the pathogenesis of hypertension. Evidence from a study by Skov et al. reported reduced diameter of afferent arterioles in spontaneously hypertensive rats [3]. Gigante et al. reported correlations in atrophic changes of the renal parenchyma with parameters associated with a reduction in renal function in hypertensive patients [4]. In addition, there is evidence for renal arterial vessel hypertrophy in human hypertension and experimental models [5]. Renal inflammation plays a key role in the progression of Chronic Kidney Disease (CKD) [6] and the development of hypertension [7]. Renal sympathetic nerves are thought to contribute to the activation of dendritic cells, T-cell infiltration, and kidney damage in the development of hypertension [8]. Pro-inflammatory cytokines, such as tumor necrosis factor-alpha (TNF-α), are known to be associated with renal inflammation and hypertension [9].

Several proteins have emerged as important markers and mediators of renal inflammation and injury. Kidney injury molecule-1 (KIM1) is a transmembrane protein upregulated during tubular epithelial cell injury and serves as a biomarker for proximal tubule damage [10,11]. Toll-like receptor 2 (TLR2) and toll-like receptor 4 (TLR4) are membrane receptors that have been found to be reduced by AAT in mouse pancreatic islet macrophages [12]. CD36, a scavenger receptor, promotes lipid uptake and oxidative stress in renal tissues, exacerbating inflammation and fibrosis [13]. Similarly, CD93 has been implicated in modulating inflammatory signaling, such as through nuclear factor-kappa B (NF-Kappa B) [14]. STAT3 is a crucial signaling molecule in tubular epithelial cells that prevents the progression of nephropathy and kidney fibrosis [15].

Elevated sodium retention and the development of hypertension have been previously shown to be mitigated by alpha1 antitrypsin (AAT) [16,17]. AAT is a serine protease inhibitor that has been shown to have anti-inflammatory properties in experimental animal disease models [18,19,20]. Emerging evidence suggests that heat shock proteins (HSP), particularly HSP70, may be protective against inflammation [21]. HSP70, a stress-inducible molecular chaperone, plays a role in cellular homeostasis and has demonstrated renoprotective effects in models of kidney injury, enhancing cellular resilience under inflammatory and stress conditions [21,22]. HSP70’s promotion of regulatory T-cell (Treg) activation plays an important role in the control of renal inflammation and hypertension. This mechanism indicates potential therapeutic implications of HSP70 in the regulation of immune responses [23]. At the same time, HSP70 is known to inhibit the activation of the apoptosis-related protein Bax [24], inhibit activation of the NLRP3 inflammasome [25], and reduce mitochondrial dysfunction [25]. Furthermore, HSP70 decreases pro-inflammatory cytokine production by inhibiting NF-Kappa B pathways, a critical mechanism in the control of inflammation [26].

The goal of this study was to test our hypothesis that the administration of purified hAAT can mitigate hypertension induced by salt-loading in otherwise healthy kidneys by reducing renal inflammation in a mechanism involving an increase in HSP70 and a decrease in the expression of specific transcription factors and cell surface receptors.

## 2. Materials and Methods

### 2.1. Animals and Treatments

129Sv wild-type mice (Jacksons Laboratory; Bar Harbor, ME, USA) were individually housed in metabolic cages and were kept on a normal salt diet (0.4% NaCl) (Teklad, Envigo, Indianapolis, IN, USA) for 5 days and then salt-loaded (4% NaCl) (Teklad, Envigo) for 11 days to induce hypertension. These 129Sv mice develop hypertension after 7–10 days of salt-loading. After the 11th day, the mice were either administered clinical grade hAAT (Prolastin^®^C, (Grifols Therapeutics Inc., Research Triangle Park, NC, USA)), hAAT (2 mg/mouse/every other day) or the vehicle (0.9% sterile saline (Fisher Scientific) via intraperitoneal (IP) injection every other day, for a total of 3 injections after the 11th day of salt-loading. The male mice were 4 weeks old at the start of the study and 4 mice were given the vehicle treatment while 5 mice were given the hAAT treatment. The mice were euthanized on the 24th day of the study. The duration of the study was 24 days. All animal studies were performed under an approved University of Florida’s Institutional Animal Care and Use Committee protocol (#IACUC202300000702 approved 5/15/2024).

### 2.2. Blood Pressure Measurements

Blood pressure was measured by the tail-cuff method (IITC MRBP System from Life Science Inc.) twice a day and the data were analyzed using the MRBP Software.

### 2.3. Urinary Creatine Measurements

A creatinine assay kit (ab204537) (Abcam; Waltham, MA, USA) was used to measure urinary creatinine levels while following the manufacturer’s instructions.

### 2.4. SDS-PAGE, Western Blotting, and Densitometric Analysis

Kidney cortex lysates were homogenized, and a bicinchoninic acid (BCA) protein assay (ThermoFisher Scientific; Waltham, MA, USA) was performed according to the manufacturer’s instructions. The soluble lysate samples were loaded into 20-well gels after total protein concentrations were determined. For each gel, 50 µg of total protein was resolved on a Criterion electrophoresis system (BioRad; Hercules, CA, USA) at 200 V for one hour and then were transferred to nitrocellulose membranes (ThermoFisher Scientific) in Towbin buffer at 100 V for half an hour. For another hour, the membranes were blocked with a 5% non-fat milk 1x TBS solution. The blots were then washed with 1x TBS solution and incubated in primary antibodies (Table 1) at a dilution of 1:1000 at 4 degrees Celsius overnight. Depending on the antibody, either goat anti-rabbit or goat anti-mouse secondary antibody (BioRad), prepared at a 1:3000 dilution in blocking solution, was incubated with the membranes for 1 h. The membranes were then washed three times with 1x TBS and then incubated with ECL reagent (BioRad) for seven minutes before being imaged using an iBright gel documentation system (ThermoFisher). Densitometry of the immunoreactive bands was quantified using ImageJ software version 1.53 (National Institutes of Health, Bethesda, MD, USA) [27].

### 2.5. Immunofluorescence

Formalin-fixed paraffin-embedded kidneys were cut into 4 μm sections and underwent 2 exchanges of xylene and a series of exchanges of ethanol dilutions. After an exchange with type 1 water for 3 min, the slides were put in a boiling citrate buffer for 20 min, washed in type 1 water for 3 min, and placed in 1x PBS for 5 min. The tissues were blocked with 2.5% normal horse serum (Vector Laboratories; Newark, CA, USA) then incubated for 30 min before being incubated in primary antibody prepared at a 1:500 dilution in blocking solution for an hour. After being washed with 1x PBS, the tissues were incubated with VectaFluor Duet Reagent and then a drop of Vectashield anti-fade mounting media (Vector Laboratories) was applied before the slides were cover slipped. The tissues were imaged for fluorescence on an Olympus microscope using a 40x objective.

### 2.6. Statistical Analysis

Sigmaplot 15.0 software (Systat Software, San Jose, CA, USA) was used to perform statistical analysis. A Student’s *t*-test was performed to make comparisons between the two groups. A *p*-value of less than 0.05 was considered to be statistically significant.

## 3. Results

### 3.1. hAAT Reduces Systolic Blood Pressure in Salt-Loaded Hypertensive 129Sv Mice

Salt-loading of 129Sv mice resulted in the development of hypertension (Table 2). The administration of hAAT, compared to the vehicle treatment, significantly reduced blood pressure (Table 2). Urinary creatinine and urinary sodium excretion were comparable between the groups (Table 2).

### 3.2. hAAT Attenuates Renal KIM1 Protein Expression in the Kidney of Salt-Loaded 129Sv Mice

**Figure 1:**
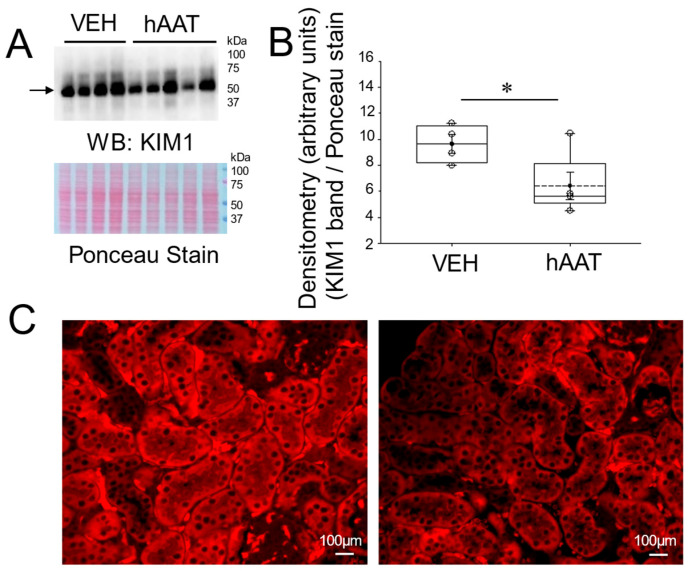
KIM1 protein expression in kidney cortex fractions of salt-loaded 129Sv mice administered hAAT or VEH. (**A**) Western blot (top) of KIM1 protein expression in kidney cortex lysates from 129Sv mice administered hAAT or VEH. Ponceau stain (bottom) used to assess lane loading, (**B**) Densitometric analysis of the immunoreactive KIM1 band in the Western blot in panel (**A**). (**C**) immunohistochemistry analysis of KIM1 protein expression in the kidneys of 129Sv mice administered VEH (left) or hAAT(right). The arrow indicates the band in the Western blot used for densitometry. N = 4 VEH treated mice and n = 5 hAAT treated mice. hAAT refers to human alpha-1 antitrypsin; VEH refers to vehicle. * refers to a *p*-value < 0.05. Original images can be found in Supplementary Materials.

Kidney injury molecule 1 (KIM1) is commonly used as an indicator of tubular cell injury [28]. To investigate whether the administration of purified hAAT could mitigate tubular cell injury in the kidney cortex of salt-loaded hypertensive 129Sv mice, we measured KIM1 protein expression by immunoassay. As shown in the Western blot and densitometric analysis in Figure 1A,B and in the immunohistochemistry analysis in Figure 1C, hAAT significantly reduced KIM1 protein expression levels within the kidneys of 129Sv mice when compared to vehicle-treated hypertensive mice.

### 3.3. hAAT Attenuates Expression of Inflammatory Signaling Proteins in the Kidney of Salt-Loaded 129Sv Mice

A previous study by our group showed the administration of purified hAAT significantly reduced diacylglycerol levels and protein kinase C (PKC) activity in 129Sv mice to mitigate hypertension during both the active and inactive cycles [29]. Here, we aimed to investigate whether the blood-pressure-lowering effects of hAAT are in part due to anti-inflammatory and renal-protective mechanisms.

**Figure 2:**
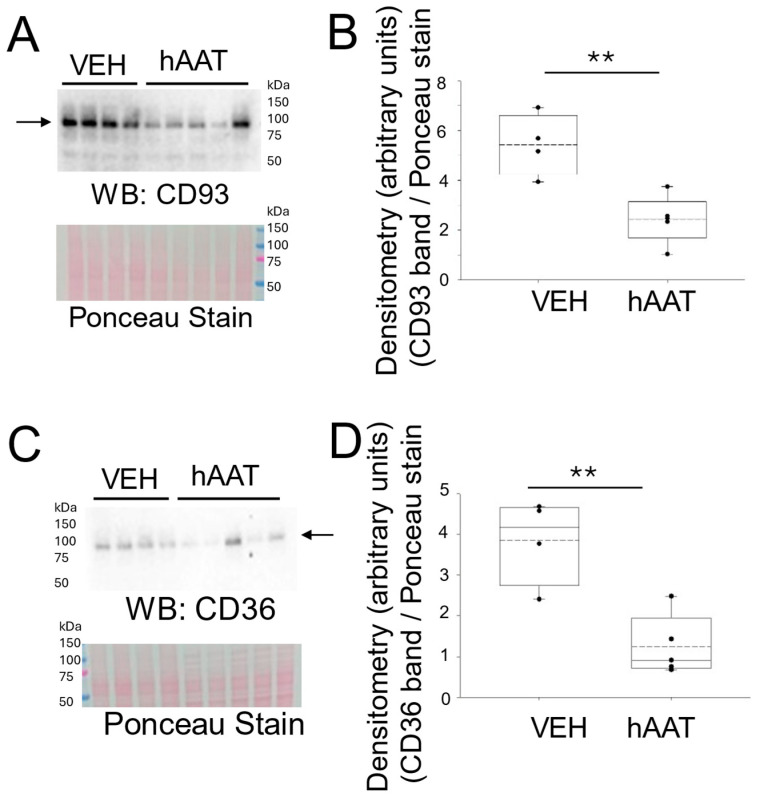
CD93 and CD36 protein expression in kidney cortex fractions of 129Sv mice treated with hAAT or VEH. (**A**) Western blot (top) of CD93 protein expression in each group. Ponceau stain (bottom) used to assess lane loading, (**B**) Densitometric analysis of the immunoreactive CD93 band in the Western blot in panel (**A**), (**C**) Western blot (top) of CD36 protein expression. Ponceau stain (bottom) used to assess lane loading, (**D**) Densitometric analysis of the immunoreactive CD36 band in the Western blot in panel C. Arrows indicate the band in the Western blot used for densitometry. N = 4 VEH treated mice and n = 5 hAAT treated mice. hAAT refers to human alpha-1 antitrypsin; VEH refers to vehicle. ** refers to a *p*-value < 0.01. Original images can be found in Supplementary Materials.

The cluster of differentiation 93 (CD93) is known to be expressed in endothelial cells and to be associated with inflammation [30]. In addition, an increase in membrane expression of CD93 was reported for activated macrophages [30]. Other studies have shown an increase in soluble CD93 in acute and chronic inflammation conditions [31,32]. Here we investigated whether hAAT administration could attenuate the levels of CD93 in kidney cortex fractions from hypertensive 129Sv mice. As shown in Figure 2, CD93 expression in the kidneys of 129Sv mice was attenuated in animals that received hAAT compared to the vehicle (Figure 2A,B). Since CD36 is known to promote tissue inflammation [33], we investigated whether its expression is also decreased in the kidneys of hypertensive 129Sv mice administered hAAT compared to the vehicle. Similar to the decreased expression of CD93, there was a decrease in CD36 in the kidneys of the 129SV mice given hAAT (Figure 2C,D).

### 3.4. hAAT Administration Decreases the Level of NF-Kappa B and STAT3 Protein Expression in the Kidney Cortex of Salt-Loaded 129Sv Mice

**Figure 3:**
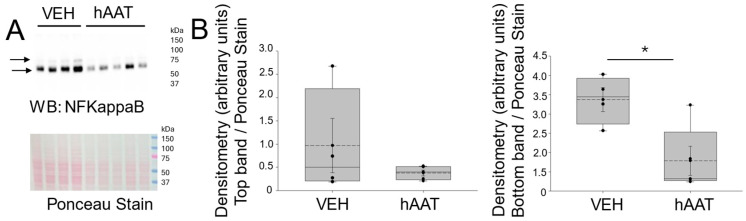
NF-Kappa B protein expression in kidney cortex fractions of 129Sv mice treated with hAAT or VEH. (**A**) Western blot (top) of NF-Kappa B protein expression in each group. Ponceau stain (bottom) used to assess lane loading, (**B**) Densitometric analysis of the immunoreactive NF-Kappa B band in the Western blot in panel (**A**). Arrows indicate the band in the Western blot used for densitometry. N = 4 VEH treated mice and n = 5 hAAT treated mice. hAAT refers to human alpha-1 antitrypsin; VEH refers to vehicle. * refers to a *p*-value < 0.05. Original images can be found in Supplementary Materials.

**Figure 4:**
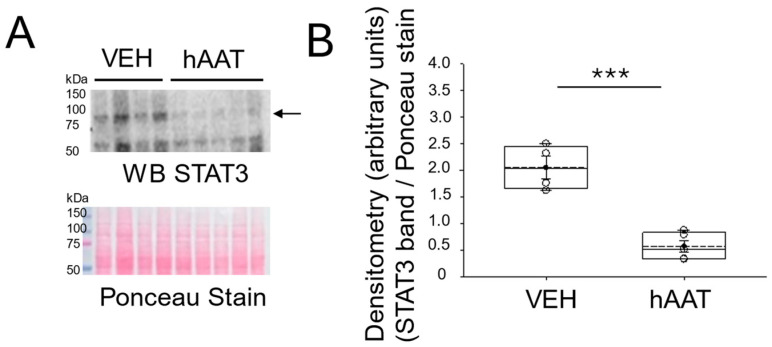
STAT3 protein expression in kidney cortex fractions of 129Sv mice treated with hAAT and vehicle. (**A**) Western blot (top) of STAT3 protein expression in each group. Ponceau stain (bottom) used to assess lane loading, (**B**) Densitometric analysis of the immunoreactive STAT3 band in the Western blot in panel (**A**). Arrow indicates the band in the Western blot used for densitometry. N = 4 VEH treated mice and n = 5 hAAT treated mice. hAAT refers to human alpha-1 antitrypsin; VEH refers to vehicle. *** refers to a *p*-value < 0.001. Original images can be found in Supplementary Materials.

Since previous studies have shown both the NF-Kappa B and STAT3 signaling pathways are linked to inflammation associated with various diseases, we investigated whether the administration of hAAT could reduce abnormally high levels of these proteins in the kidneys of hypertensive 129Sv mice. First, the Western blot and densitometric analysis of pNF-Kappa B (p65) showed a significant decrease in the levels of this protein in the kidney cortex of salt-loaded 129Sv mice administered hAAT when compared to the vehicle treatment (Figure 3). Next, a similar trend in STAT3 protein expression was seen in the kidney of salt-loaded 129Sv mice administered hAAT compared to the vehicle (Figure 4).

### 3.5. Purified hAAT Reduces TLR2/4 Expression in the Kidney of Salt-Loaded 129Sv Mice

**Figure 5:**
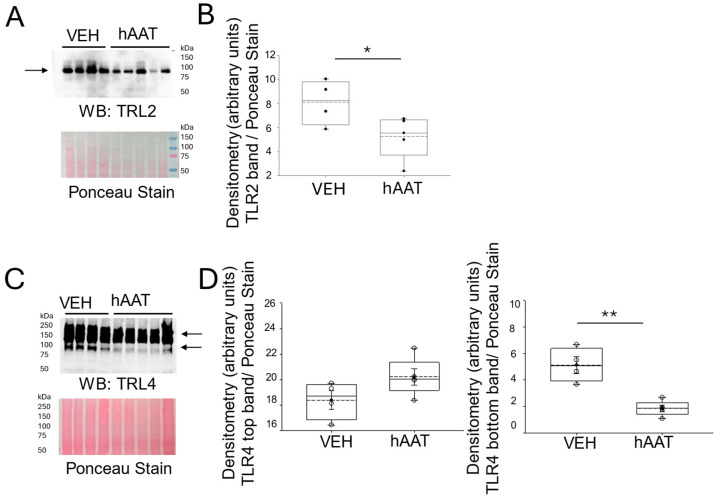
TLR2 protein expression in kidney cortex fractions of 129Sv mice treated with hAAT and vehicle. (**A**) Western blot (top) of TLR2 protein expression in each group. Ponceau stain (bottom) used to assess lane loading, (**B**) Densitometric analysis of the immunoreactive TLR2 band in the Western blot in panel (**A**). (**C**) Western blot for TLR4 protein expression in each group. Ponceau stain (bottom) to assess lane loading, (**D**) Densitometric analysis of the immunoreactive TLR4 band in the Western blot in panel (**C**). The arrow indicates the band in the Western blot used for densitometry. N = 4 VEH treated mice and n = 5 hAAT treated mice. hAAT refers to human alpha-1 antitrypsin; VEH refers to vehicle. * refers to a *p*-value < 0.05, ** refers to a *p*-value < 0.01. Original images can be found in Supplementary Materials.

Since toll-like receptor 2 (TLR2) is known to be involved in inflammatory responses and induce migration of neutrophils and macrophages [34], we investigated whether hAAT affected its expression in the kidneys of salt-loaded 129Sv mice. As shown by the Western blot and densitometric analysis in Figure 5A,B, the administration of purified hAAT decreased TLR2 protein expression in the kidneys of salt-loaded 129Sv mice. Similarly, TLR4 protein expression was decreased in the kidneys after hAAT administration, as shown in Figure 5C,D.

### 3.6. Purified hAAT Augments HSP70 Protein Expression in the Kidney of Salt-Loaded 129Sv Mice

**Figure 6:**
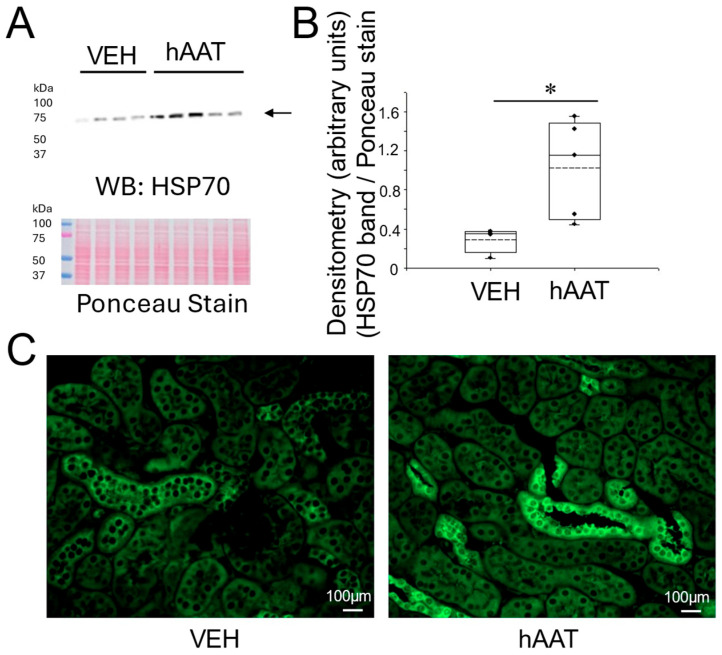
HSP70 protein expression in kidney cortex fractions of 129Sv mice treated with hAAT and vehicle. (**A**) Western blot (top) of HSP70 protein expression in each group. Ponceau stain (bottom) used to assess lane loading, (**B**) Densitometric analysis of the immunoreactive HSP70 band in the Western blot in panel (**A**). (**C**) immunohistochemistry analysis of HSP70 protein expression. The arrow indicates the band in the Western blot used for densitometry. N = 4 VEH treated mice and n = 5 hAAT treated mice. hAAT refers to human alpha-1 antitrypsin; VEH refers to vehicle. * refers to a *p*-value < 0.05. Original images can be found in Supplementary Materials.

Since the multifunctional protein HSP70 is known to be involved in maintaining cellular homeostasis and reducing organ dysfunction [35], we investigated whether the administration of purified hAAT could increase HSP70 expression in the kidneys of 129Sv mice that were salt-loaded to induce hypertension. As shown by the Western blot and immunohistochemistry, the administration of hAAT significantly augmented HSP70 protein expression in the kidney of these mice compared to vehicle-treated mice (Figure 6).

### 3.7. Administration of hAAT Augments IGF-1 Protein Expression in the Kidney of 129Sv Mice

**Figure 7:**
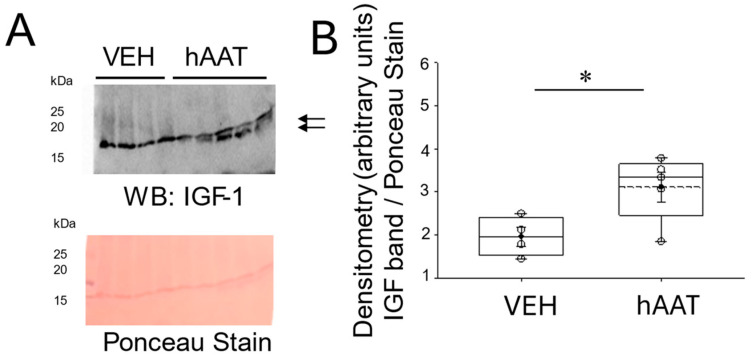
Insulin growth factor-1 protein expression in kidney cortex fractions of 129Sv mice treated with hAAT and vehicle. (**A**) Western blot (top) of IGF-1 protein expression in each group. Ponceau stain (bottom) used to assess lane loading, (**B**) Densitometric analysis of the immunoreactive IGF-1 band in the Western blot in panel (**A**). The arrow indicates the band in the Western blot used for densitometry. N = 4 VEH treated mice and n = 5 hAAT treated mice. hAAT refers to human alpha-1 antitrypsin; VEH refers to vehicle. * refers to a *p*-value < 0.05. Original images can be found in Supplementary Materials.

To investigate whether ATT exerts longevity effects in concert with anti-inflammation, we measured changes in insulin-like growth factor (IGF-1) protein expression in salt-loaded 129Sv mice Administered vehicle or hAAT. The Western blot and densitometric analysis showed an increase in IGF-1 protein expression in the kidneys of mice administered hAAT compared to mice administered VEH (Figure 7).

## 4. Discussion

In this study, we showed for the first time that the administration of purified hAAT to salt-loaded 129Sv mice resulted in the reduction of protein expression of multiple inflammatory transcription factors and signaling proteins (Figure 7). We chose 129Sv mice because these mice have been previously shown to be salt-sensitive and develop hypertension upon salt-loading. We used the same dose of purified hAAT based on that used in other animal studies to mitigate pathophysiology associated with salt-induced hypertension.

KIM1-positive staining is found in tubular epithelial cells [11] and in glomerular cells [36]. In this study, we show for the first time that hAAT administration significantly reduced KIM1 protein expression in the kidneys of salt-loaded 129Sv mice. Similarly, in a study by Maicas et al. that evaluated the effects of hAAT administration in a renal ischemic reperfusion (I/R) injury model, KIM1 levels in the urine were shown to be decreased [37]. Both the Maicas et al. study and our study support the hypothesis that hAAT mitigates kidney injury through immunomodulation, but our findings suggest a distinct mechanism in chronic hypertension through key transcription factors along with alterations in CD93 and HSP70 expression pathways not explored in the I/R injury model. A study by Han et al. investigated KIM1 protein expression in normal human kidney and acute tubular necrosis, and concluded that KIM1 may serve as a useful biomarker for human renal proximal tubule injury [10]. Additionally, Kadioglu et al. reported that increased levels of KIM1 are positively correlated with kidney injury in hypertensive nephropathy, acute kidney injury, and duration of disease severity [38]. This supports the use of KIM1 as a marker in our model and highlights hAAT’s potential as a therapeutic agent in hypertensive kidney injury.

We also showed for the first time that hAAT administration significantly reduced TLR2 and TLR4 protein expression in the kidneys of salt-loaded 129Sv mice. This aligns with the known role of TLR2 in mediating inflammatory responses and activating neutrophils and macrophages. The anti-inflammatory effects of hAAT in the kidney may be mediated by downregulating TLR2 signaling and could attenuate immune cell recruitment and activation within the kidney. This is consistent with findings from Jonigk et al., which demonstrated that purified AAT reduces inflammatory responses in elastase-deficient mice [12]. Additionally, Elshikha et al. showed that hAAT inhibits TLR9 signaling in dendritic cells, suggesting a broader role of hAAT in modulating TLR-mediated pathways [39]. The reduction in TLR2 expression may contribute to the overall anti-inflammatory and blood pressure-lowering effects of hAAT.

Both NF-Kappa B and STAT3 signaling pathways are known to be linked to inflammation associated with cancer [40,41], dry-eye disease [42], arthritis [43], septic cardiomyopathy [44], and acute kidney injury [45]. A previous study by Hong et al. reported that CD93 functions as a negative regulator of NF-Kappa B-mediated inflammatory signaling and the knockdown of CD93 promotes microglial inflammation [14]. In a study by Yuan et al., hAAT was reported to suppress NF-Kappa B activity and prolong the lifespan of Drosophila [46]. Consistent with this effect, in this study, we showed that the administration of purified hAAT reduces pNF-Kappa B (p65) in the kidneys of 129Sv mice. In addition, hAAT was found to reduce CD93 protein expression in the kidneys of salt-loaded 129Sv mice. One possible reason for the discrepancy between our study and the study by Hong et al. could be that the study was conducted using neural cells and we probed for CD93 protein expression in the kidney.

Our study demonstrated that hypertensive 129sv mice treated with hAAT exhibited decreased CD36 expression in kidney lysates. This finding aligns with previous research indicating the renoprotective effects of hAAT. Maicas et al. showed that hAAT therapy reduced renal dysfunction and acute tubular necrosis in a murine model of bilateral kidney ischemia-reperfusion injury, suggesting its potential to mitigate renal inflammation and injury [37]. Additionally, CD36 has been implicated in mediating oxidative stress and inflammation in chronic kidney disease, as evidenced by Okamura et al., who found that CD36 deficiency led to reduced fibrosis and oxidative stress in a model of CKD [13]. Moreover, CD36 is known to mediate the uptake of oxidized lipids and fatty acids, which can exacerbate renal injury through lipid accumulation and subsequent fibrosis [47]. The observed decrease in CD36 expression in our study may contribute to the anti-inflammatory and cytoprotective effects of hAAT, further supporting its therapeutic potential in renal pathologies associated with inflammation and oxidative stress.

Our findings are further corroborated by previous reports suggesting that hAAT mediates its anti-inflammatory effects via inhibition of STAT3 phosphorylation. For instance, Stanke et al. demonstrated that AAT suppressed STAT3 activation in human lung epithelial air-liquid-interface cultures [48]. While their study focused on the lungs and cystic fibrosis, our findings demonstrated the reduction of phosphorylated STAT3 in the kidneys of a salt-induced hypertensive model, adding to the growing evidence of hAAT’s potential as a therapeutic agent across multiple organ systems. STAT3 plays a significant role in the pathophysiology of renal diseases, moreover, the inhibition of STAT3 appears to be a useful strategy for mitigating renal damage. Zheng et al. found that the pharmacological blockade of STAT3, using the inhibitor S31-201, significantly reduced kidney injury markers in diabetic mice [15]. Similarly, Chen et al., showed that blocking IL-6 trans-signaling protects against fibrosis and reduces inflammation [49]. This evidence combined with our findings in the hypertensive model suggests that STAT3 activation is a critical event in renal pathophysiology and that inhibiting its activation can mitigate damage. Therefore, by demonstrating the reduction of STAT3 through hAAT administration in our salt-sensitive hypertension model, we may better understand the effects of hAAT.

In this study, we also investigated whether HSP70 was differentially expressed between the two cohorts of mice treated with vehicle or purified hAAT. One benefit of using purified hAAT over small molecular drugs is that purified hAAT is endogenous and there are not any known adverse effects. Second, hAAT is already known to have numerous beneficial effects including anti-inflammaging effects [50]. HSP70 is known for its protective effects in kidney injury. The cytoprotective role of HSP70 on angiotensin II-induced epithelial–mesenchymal transition after blockade of the AT1 receptor was reviewed by Costantino et al. [51]. Previous research demonstrated that HSP70 inhibits NF-Kappa B-mediated inflammatory pathways, reduces oxidative stress, and prevents protein misfolding. HSP70 has also been shown to inhibit apoptosis by interfering with the activation of key apoptotic proteins, such as Bax, thereby preserving renal tubular epithelial cell integrity under stress conditions [52,53]. In this study, Western blot analysis and immunohistochemistry confirmed the upregulation of HSP70 in hAAT-treated mice, further supporting the anti-inflammatory role of hAAT and positioning HSP70 as not only a biomarker of protective cellular response but also as a mechanistic link through which hAAT confers renal protection in salt-sensitive hypertension.

In light of the recent literature, particularly by Hao et al., it is important to recognize that immune cell-mediated inflammation, including activation of various macrophages, T cells, and cytokines, plays a crucial role in hypertensive kidney progression [54]. hAAT administration in our model led to normalization of blood pressure, reduction of inflammatory markers, and upregulation of HSP70. While direct targeting of HSP70 or other chaperones could be an alternative therapeutic approach, hAAT offers the advantage of acting upstream at multiple points of the inflammatory cascade. Additionally, our findings also support the dual anti-inflammatory and anti-aging properties of hAAT. Yuan et al. reported that hAAT, not only suppresses NF-Kappa B-driven inflammation but also modulates pathways linked to stress and cellular aging [50]. Our findings are consistent with this reduction in key markers, suggesting that hAAT’s actions are not solely anti-inflammatory, but also involve mechanisms that may promote cellular resilience over time. While we did not directly assess aging markers such as telomerase activity, we did show insulin-like growth factor (IGF-1)-1 protein levels are augmented in the kidney of 129Sv mice administered purified hAAT (Figure 7). Our results are consistent with the idea that hAAT’s anti-inflammatory actions may have potential anti-aging benefits, enhancing long-term tissue protection, especially in stress conditions such as salt-sensitive hypertension. Although there are multiple novel findings from this study that may help elucidate the molecular mechanisms governing the beneficial effects of hAAT in reducing hypertension, there are some limitations to our study. First, although it is known that CD36 promotes NLRP3 inflammasome activation in the kidneys of diabetic animals [55], we did not investigate changes in NLRP3 in the kidneys of salt-loaded hypertensive mice after the administration of hAAT. Second, although our study shows that the administration of purified hAAT reduces KIM1, CD93, CD63, NF-Kappa B, STAT3, and TLR2/4 levels in the kidneys of salt-loaded hypertensive 129Sv mice, future studies can incorporate the use of a recombinant protein as a control to further corroborate the effect on markers of tubular cell damage. Finally, although we did not directly investigate whether anti-inflammation mitigates oxidative stress or whether anti-oxidants mitigate inflammation, these studies are warranted in the context of the beneficial effects of hAAT in renal inflammation and pathophysiology.

## 5. Conclusions

**Figure 8:**
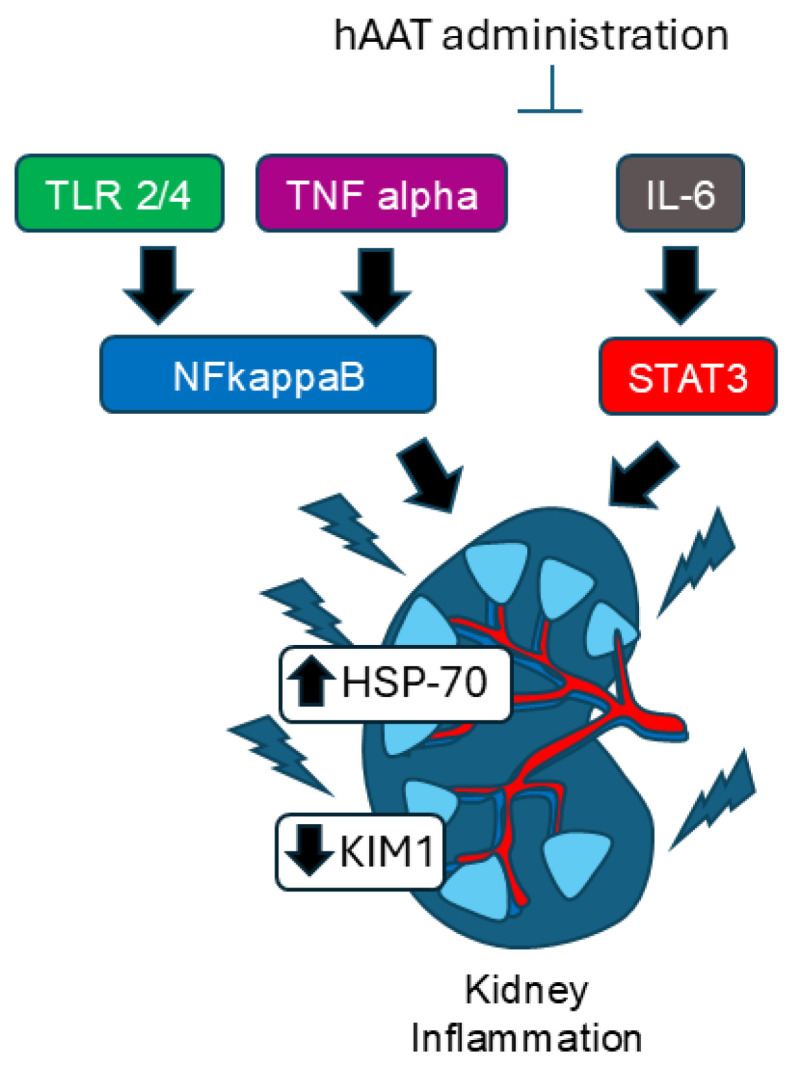
Proposed model for the protective effect of purified hAAT in mitigating renal inflammation after the development of hypertension from salt-loading. Administration of purified hAAT inhibits TLR2, NF-Kappa B, and STAT3 pathways while upregulating the expression of HSP70 protein in the kidney of 129Sv after salt-loading. As a result, the levels of KIM1 are decreased and blood pressure is normalized.

Here we show that the administration of purified hAAT mitigates renal inflammation and reduces high blood pressure induced from salt-loading. By investigating multiple inflammatory mediators (CD93, CD36, STAT3, NF-Kappa B, and TLR2/4), our results highlight the therapeutic potential of hAAT in treating kidney inflammation (Figure 8). hAAT appears to exert broad-spectrum anti-inflammatory effects. These results may make hAAT a promising candidate for the treatment of kidney-related diseases (such as acute and chronic kidney injury) accompanied by increased inflammation. Further studies are needed to elucidate the precise molecular mechanisms underlying hAAT’s effects on these pathways.

The results from this study could lead to future research directions. It will be important to evaluate whether hAAT treatment alters blood pressure outcomes based on time-of-day effects on transcription factor activity to understand how hAAT’s effects are influenced by circadian rhythms. Additionally, spatial localization studies of CD93, CD36, HSP70, and other key proteins could provide insight into region-specific responses within the kidney. Future studies are needed to determine the mechanism by which hAAT is taken up by renal epithelial cells. Finally, future studies may investigate the efficacy of long-term administration of hAAT beyond the 2–3 week time frame in our study, to evaluate sustained efficacy, durability of anti-inflammatory effects, and potential benefits or risks. Together, these avenues may advance hAAT as a therapeutic strategy for kidney and inflammatory diseases.

## Figures and Tables

**Table 1:** Antibodies used in this study.

Antibody	Company	Catalog Number
HSP70	Cell Signaling Tech (Danvers, MA, USA)	4872P
STAT3	Abcam (Waltham, MA, USA)	ab76315
TIM1/KIM1	Novus Biologicals (Centennial, CO, USA)	NBP1-76701
CD93	Santa Cruz Biotech (Dallas, TX, USA)	SC-365172
CD36	Santa Cruz Biotech	SC-7309
TLR2	Santa Cruz Biotech	SC-166900
NF-Kappa B p65	Protein Tech (Rosemont, IL, USA)	80979-1-RR

All antibodies were stored and used according to the manufacturer’s instructions.

**Table 2:** Physiological parameters of hypertensive 129Sv mice administered hAAT or veh.

Physiological Parameter	hAAT Administration	VEH Administration	*p*-Value
Systolic blood pressure (mmHg)	118.9 +/− 1.24	149.12 +/− 7.76	0.003
Urine creatinine (mg/dL)	7.47 +/− 1.05	6.01 +/− 1.94	0.506
Urinary sodium excretion (mEq/24-h)	1.14 +/− 0.34	0.45 +/− 0.042	0.124

hAAT refers to purified human alpha-1 antitrypsin. VEH refers to vehicle administration. Parameters with a *p*-value < 0.05 were considered statistically significant.

## Data Availability

Raw datasets will be made available by the corresponding author upon reasonable request.

## Supplementary Materials

The following supporting information can be downloaded at: https://www.mdpi.com/article/10.3390/biom15070951/s1, Figure S1–S7: Original images.

## Abbreviations

The following abbreviations are used in this manuscript:

AAT	alpha-1 antitrypsin
TLR	toll-like receptor
HSP	heat shock proteins
IL-6	interleukin-6
MDPI	multidisciplinary Digital Publishing Institute
TNF-α	tumor necrosis factor-alpha
CD	cluster of differentiation
KIM-1	kidney injury molecule
STAT3	signal transducer and activator of transcript 3
hAAT	human alpha-1 antitrypsin
CKD	chronic kidney disease
BP	blood pressure
