# Supplementary material for: Administration of Purified Alpha-1 Antitrypsin in Salt-Loaded Hypertensive 129Sv Mice Attenuates the Expression of Inflammatory Associated Proteins in the Kidney
Source: Biomolecules. 2025 Jun 30;15(7):951. doi: 10.3390/biom15070951 (PMC12293082; doi:10.3390/biom15070951)
Supplement: Supplementary file 1 [file biomolecules-15-00951-s001.zip › biomolecules-3586320-supplementary.pdf]

Figure S1

A

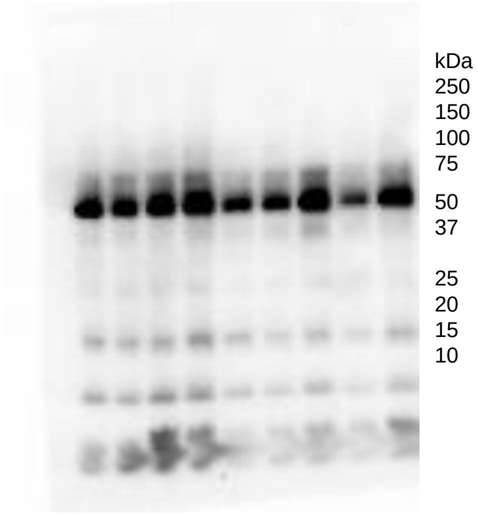

WB: KIM1

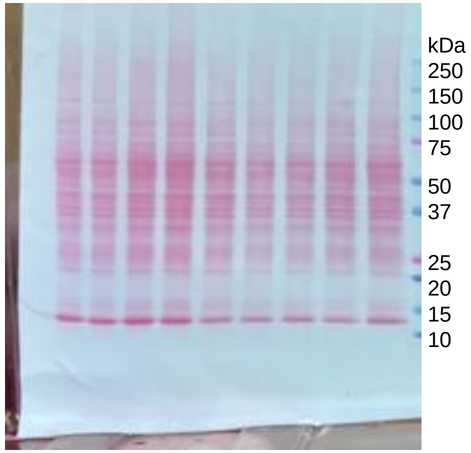

Ponceau Stain

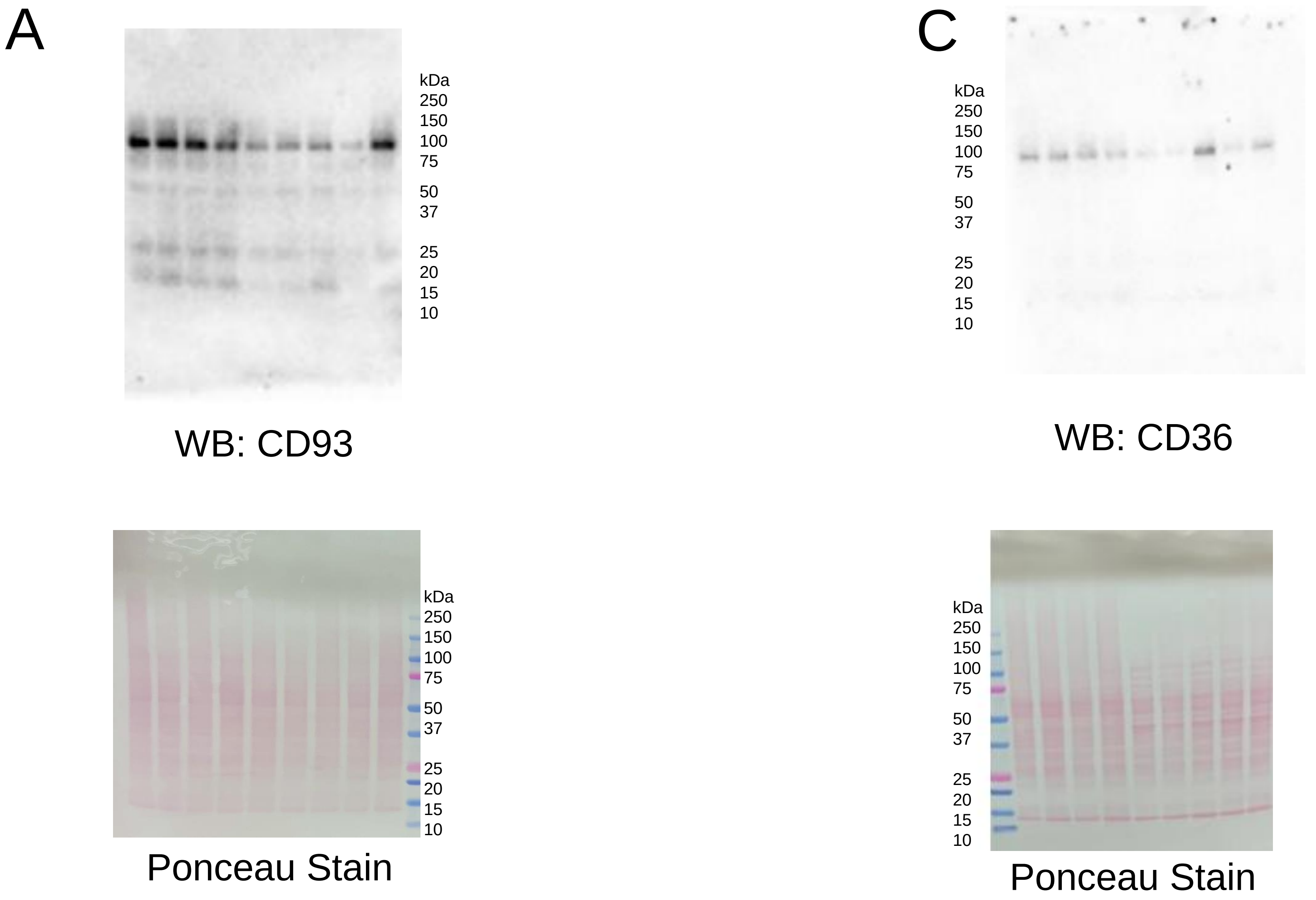
A
kDa
250
150
100
75
50
37
25
20
15
10
WB: CD93
kDa
250
150
100
75
50
37
25
20
15
10
Ponceau Stain
C
kDa
250
150
100
75
50
37
25
20
15
10
WB: CD36
kDa
250
150
100
75
50
37
25
20
15
10
Ponceau Stain

A

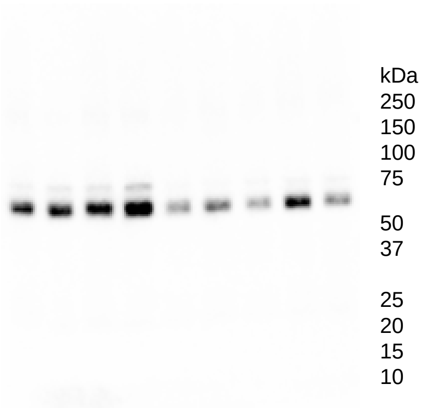

WB: NF-KappaB

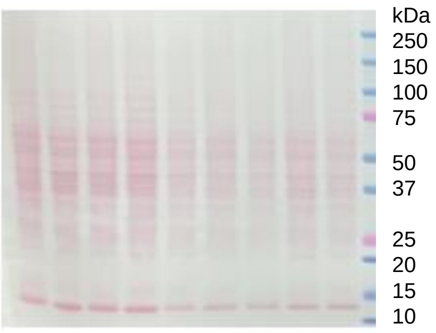

Ponceau Stain

Figure S4

A

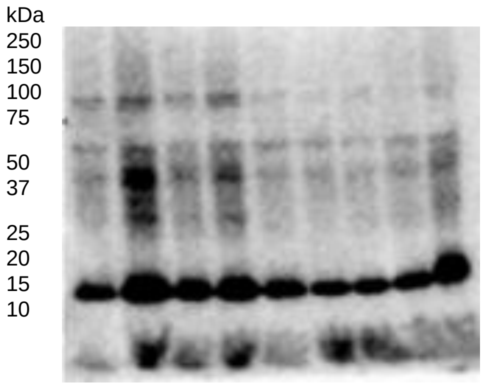

WB STAT3

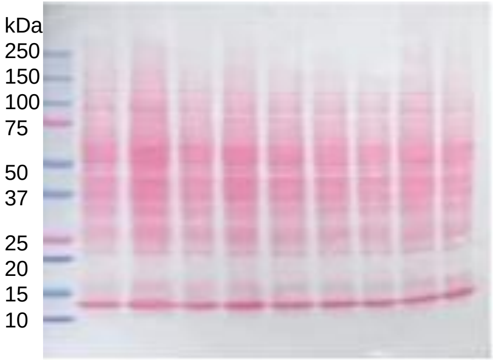

Ponceau Stain

Figure S5

A

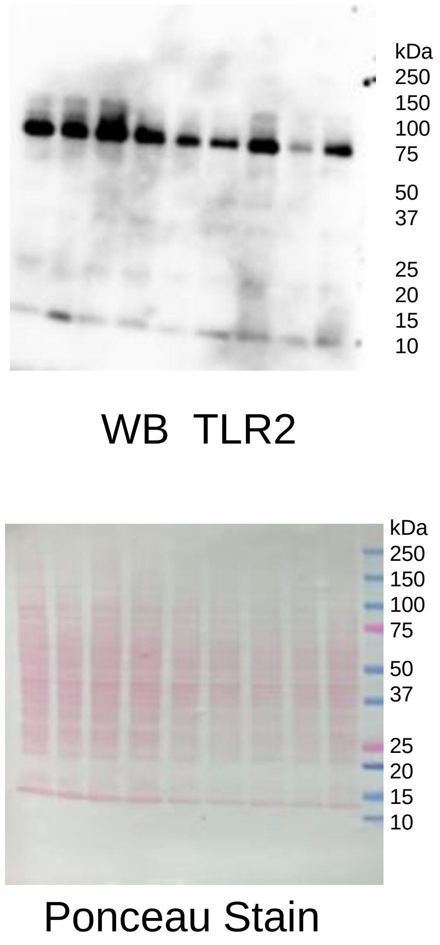
kDa
250
150
100
75
50
37
25
20
15
10
WB TLR2
kDa
250
150
100
75
50
37
25
20
15
10
Ponceau Stain

C

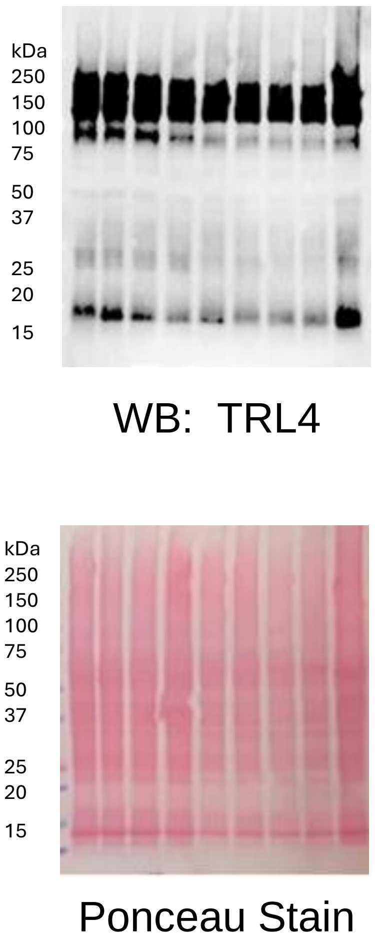
kDa
250
150
100
75
50
37
25
20
15
WB: TRL4
kDa
250
150
100
75
50
37
25
20
15
Ponceau Stain

Figure S6

A

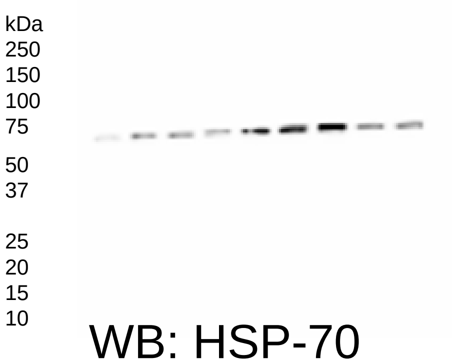

WB: HSP-70

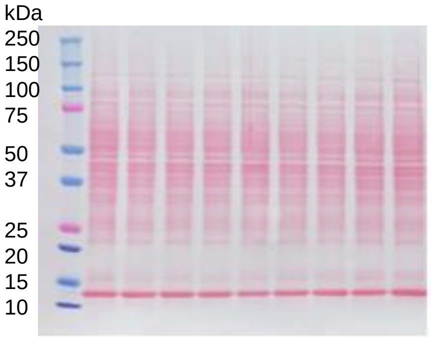

Ponceau Stain

Figure S7

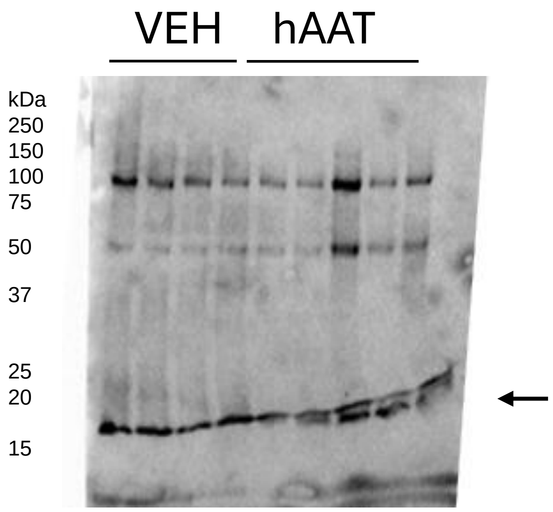

WB: IGF-1

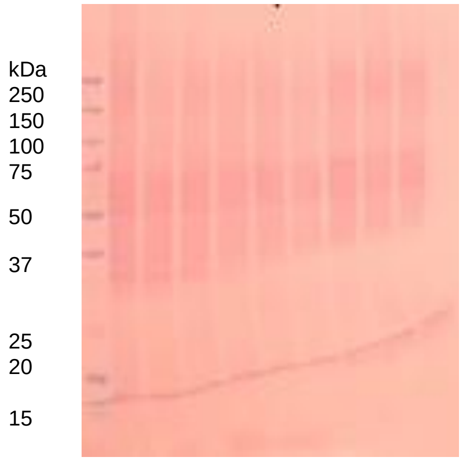

Ponceau Stain
